# Therapeutic concentrations of antibiotics inhibit Shiga toxin release from enterohemorrhagic *E. coli* O104:H4 from the 2011 German outbreak

**DOI:** 10.1186/1471-2180-12-160

**Published:** 2012-08-01

**Authors:** Diana Corogeanu, Ruben Willmes, Martina Wolke, Georg Plum, Olaf Utermöhlen, Martin Krönke

**Affiliations:** 1 Institute for Medical Microbiology, Immunology and Hygiene, Medical Center, University of Cologne, Goldenfelsstrasse 19-21, Cologne, D-50935, Germany; 2 Center for Molecular Medicine University of Cologne, Cologne, Germany; 3 German Center for Infection Research (DZIF), Cologne, Germany

**Keywords:** Shiga toxin producing *E. coli* (STEC), Enterohemorrhagic *E. coli* (EHEC), Antibiotics

## Abstract

**Background:**

The shiga toxin-producing *E. coli* (STEC) O104:H4 caused a major outbreak in Germany in spring 2011. STEC are usually susceptible to common antibiotics. However, antibiotic treatment of STEC-infected patients is not recommended because STEC may enhance production and release of shiga toxins (STX) in response to antibiotics, which eventually enhances the frequency and severity of clinical symptoms, including haemolytic uraemic syndrome (HUS) and fatalities.

**Results:**

We characterized the response to antibiotics of STEC O104:H4 isolates from two HUS patients during the German STEC outbreak in spring 2011 in comparison to the common STEC O157:H7. Liquid cultures of STEC O157:H7 and O104:H4 were incubated with graded dilutions of the antibiotics ciprofloxacin, meropenem, fosfomycin, gentamicin, rifampicin, and chloramphenicol. At defined times of antibiotic treatment, transcriptional activation of the STX2 gene, contents of STX and STX-activity in the culture supernatants were quantified. Unlike the common serotype O157:H7, STEC O104:H4 does not release STX in response to therapeutic concentrations of ciprofloxacin, meropenem, fosfomycin, and chloramphenicol.

**Conclusions:**

In future outbreaks, the response of the respective epidemiologic STEC strain to antibiotics should be rapidly characterized in order to identify antibiotics that do not enhance the release of STX. This will eventually allow clinical studies tackling the question whether antibiotic treatment impacts on the eradication of STEC, clinical course of disease, and frequency of carriers.

## Background

During the outbreak of a shiga toxin (STX) producing E. coli (STEC), strain O104:H4, in Germany between mid May and early July 2011, 3842 infected patients were reported of whom 855 developed a haemolytic-uremic syndrome (HUS) and 53 died [1]. In the light of outbreaks of STEC transmitted by contaminated food at unpredictable intervals all over the world, these recent numbers underline the serious threat posed by STEC to public health even in highly developed countries.

For the treatment of STEC-infected patients, a causal therapy to prevent the development of HUS is not available. Most importantly, the use of antibiotics is controversially discussed due to the particular response of STEC. According to the prevailing view, the use of antibiotics against STEC should be avoided because it is assumed to increase the risk of developing HUS (for review[2]). Although growth of given STEC strains is susceptible to inhibition by specific antibiotics, the bacteria may respond with enhanced release of shiga toxin activity [3,4]. High hopes rest on new therapeutic concepts aiming at binding and inactivating shiga toxin in the patient (for review [2,5]). However, these approaches are not yet clinically available and applicable.

The recent STEC outbreak prompted us to revisit the effects of antibiotics on STEC. These effects have been studied intensively in the most common STEC serotype O157:H7 that emerged as a human pathogen in 1982 [6]. Treatment of this STEC strain with antibiotics, specifically with those interfering with DNA replication, activates the SOS response of the bacteria [7]. This in turn activates the lytic cycle of the bacteriophages that encode, among others, the shigatoxin genes. Consequences are, first, the increased production of STX and, second, phage-induced lysis of *E. coli* host cells eventually resulting in the release of large amounts of STX. The influence of antibiotic treatment upon the clinical course including the frequency of HUS within the cohort of STEC-infected patients had been assessed mostly in retrospective studies [8,9]. So far, neither observations during outbreaks nor controlled clinical trials provided resilient evidence whether early and consequent antibiotic treatment of STEC-infected individuals might be effective to reliably abort the release of STX thereby preventing the development or aggravation of HUS. Notably, clinical observations as well as most studies *in vitro* focussed on O157:H7, being the most frequent serotype of STEC. This study aimed at characterizing the response of STEC O104:H4 to antibiotics with regard to the release of shiga toxin activity into the supernatants of *in vitro* cultures. Since strain O104:H4 differs genotypically and phenotypically from classical STEC, we compared its responses to antibiotics with that of the common STEC strain O157:H7.

## Results

### Susceptibility of the growth of STEC strains to select antibiotics *in vitro*

This study characterizes the response to antibiotic treatment of two isolates, P5711 and P5765, of STEC serotype O104:H4 of the German outbreak in 2011 in comparison to the most common STEC reference strain serotype O157:H7, from the National Reference Centre for Salmonella and other bacterial pathogens causing enteritis, Robert-Koch-Institute, and to the shigatoxin-negative *E. coli*, ATCC 25922.

The minimal inhibitory concentrations (MIC) for the two isolates of O104:H4, P5711 and P5765, of the antibiotics ciprofloxacin, meropenem, fosfomycin, gentamicin, rifampicin, and chloramphenicol were inconspicuous when compared to the common STEC strain O157:H7 or the STX-negative strain *E. coli* ATCC 25922 (Table 1).

**Table 1:** Minimal inhibitory concentrations of select antibiotics for two isolates of STEC strain H104:H4, STEC O157:H7, and *E. coli* ATCC 25922

	**E. coli strain**			
	**O104:H4**		**O157:H7**	**ATCC25922**
	**Isolate**			
	**P5711**	**P5765**		
**Antibiotic**	**MIC [mg/l]**^**1**^			
Ciprofloxacin	0.125	0.125	0.064	0.032
Chloramphenicol	4.0	4.0	8.0	6.0
Meropenem	0.047	0.047	0.032	0.032
Gentamicin	2.0	2.0	4.0	6.0
Rifampicin	32.0	32.0	16.0	12.0
Fosfomycin	0.25	0.25	0.094	0.19

^1^ Minimal inhibitory concentrations (MIC) were determined as described in Methods. Values depict the respective minimal concentration of a given antibiotic that inhibited the visible growth (E-test, BioMerieux).

### Transcription of the STX2 gene in STEC strains in response to treatment with antibiotics

**Figure 1:**
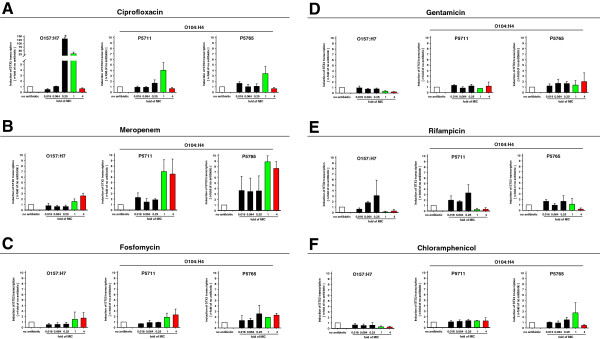
**Transcriptional induction of the STX2 gene in STEC strains O157:H7 and O104:H4 by various antibiotics.** STEC strains O157:H7 and O104:H4 were inoculated into L-broth at a density of 1x10^8^ bacteria/ml. The cultures were either left without antibiotics or treated immediately with the indicated n-folds of the MIC of the indicated antibiotics and incubated at 37°C under vigorous shaking. After 2 h, 200 μl of the bacterial suspensions were harvested to prepare total RNA and to determine by qRT-PCR the numbers of STX2-specific transcripts. Green or red columns highlight the values after treatment with the 1-fold or 4-fold MIC, respectively. This colour code is used throughout the manuscript. Shown are the means and standard errors of three independent experiments. Statistical significance is indicated by asterisks: * for p < 0.05.

Treatment of STEC with specific antibiotics may rapidly induce a SOS-response starting the lytic cycle of the bacteriophages associated with the transcription of genes coding for shiga toxins (reviewed in [7]). This may result in enhanced production and release of shiga toxins. This apprehended adverse reaction led to the recommendation to refrain from antibiotic treatment during the recent epidemic with STEC O104:H4 in Germany. Subinhibitory concentrations of antibiotics assumed to be present during the early phase of treatment, often lead to the induction of shiga toxin production [3,4]. Therefore, the mRNA coding for shiga toxin 2 was quantified at 2 h after treatment of fluid phase cultures of STEC O157:H7 and O104:H4 with graded concentrations of antibiotics. Ciprofloxacin at 0.25x MIC and 1x MIC induced STX2-transcripts about 125- and 30-fold, respectively, in the control STEC O157:H7 (Figure 1A). In sharp contrast, O104:H4 responded to 1x MIC of ciprofloxacin only by an about 3- to 4-fold increase in STX2-transcripts. Ciprofloxacin at 4x MIC reduced in both STEC O104:H4 isolates numbers of STX2-transcripts below that of untreated controls. These data reveal a remarkable difference of various strains of STEC in the transcriptional activity of the STX2-specific gene in response to graded concentrations of ciprofloxacin.

Meropenem at subinhibitory and 1xMIC did not increase the number of STX2-specific transcripts in STEC O157:H7 (Figure 1B). Similarly, subinhibitory MIC of meropenem did not enhance the STX2-transcripts in STEC O104:H4. At 4x MIC meropenem enhanced the numbers of STX2-specific transcripts only about 2.5-fold in STEC O157:H7. In contrast, both isolates of STEC O104:H4 responded a little stronger than O157:H7 to the 1x and 4x MIC with about 7- to 9-fold increased numbers of STX2-specific transcripts (Figure 1B). None of these increases was statistically significant. Nevertheless, these data in comparison with the response to ciprofloxacin (Figure 1A) suggest that strain-specific and antibiotics-specific responses of STEC should be carefully characterized.

In both strains O157:H7 and O104:H4, fosfomycin at the 1x and 4x MIC slightly increased the numbers of STX2-specific mRNA up to 2-fold (Figure 1C).

Treatment with gentamicin resulted in a dose dependent gradual reduction of STX2-specific transcripts in cultures of STEC strain O157:H7 and had no consistent effect on strain O104:H4 (Figure 1D).

Up to 0.25x MIC, rifampicin dose-dependently increased the numbers of STX2-specific transcripts in both STEC O157:H7 and O104:H4 (Figure 1E), whereas 1x and 4x MIC of rifampicin reduced the abundance of STX2-specific mRNA below levels in untreated bacteria.

STEC O157:H7 responded to the 1x and 4x MIC of chloramphenicol with more than 50% reductions of the numbers of STX2-specific mRNA (Figure 1F). In STEC O104:H4 chloramphenicol did not affect the number of STX2-specific transcripts.

These data indicate that two independent isolates, P5711 and P5765, of STEC O104:H4 respond during the first 2 h of treatment with specific antibiotics concordantly with regard to the induction of the transcription of the gene coding for the shiga toxin STX2. It is important to note that STEC O104:H4 and O157:H7 respond in a diverging manner to specific antibiotics. Specifically, ciprofloxacin induced STX2-transcripts in vast amounts in strain O157:H7 but it had only marginal effects on strain O104:H4. In contrast, meropenem at 1x MIC and 4x MIC induced strain O104:H4 to transcribe enhanced numbers of STX2-transcripts, but not in strain O157:H7. The other antibiotics used in this study had either no or only marginal effects on the numbers of STX2-transcripts during the first 2 h of antibiotic treatment.

### Release of shiga toxin into the supernatants by treatment of STEC strains with antibiotics

Antibiotics could induce the release of preformed STX2 and/or of STX2 newly synthesized from induced STX2-mRNA transcripts. Therefore, both the contents and the toxin activity of shiga toxins in the supernatants of fluid phase cultures were measured after cultivation of STEC for 24 h in the presence of graded concentrations of antibiotics. The shiga toxin contents of the supernatants of STEC cultures were measured with a commercially available EIA that detects both shiga toxins 1 and 2. Notably, STEC O104:H4 produces only shiga toxin 2 [10], while STEC O157:H7 produces both shiga toxins 1 and 2 [11].

STEC O157:H7 responded to lower concentrations of ciprofloxacin with a pronounced release of shiga toxins. A 0.064x MIC led to 32-fold higher titers and 0.25x MIC and 1x MIC, respectively, led to 512- and 256-fold higher titers than those of untreated controls. The 4x MIC increased the titers still 32-fold. In cultures of STEC O104:H4, ciprofloxacin at 0.25x MIC and 1x MIC, respectively, led to 32- and 256-fold higher titers of shiga toxin than in untreated controls. Treatment with 4x MIC of ciprofloxacin resulted in titers slightly below those of controls. These data confirm previous reports about the strongly increased release of shiga toxin by STEC O157:H7 in response to ciprofloxacin [4]. Compared to STEC O157:H7, the response characteristics of STEC O104:H4 are clearly attenuated as shown both by lower titers of STX2 in response to subinhibitory MIC and by completely abolished release of shiga toxin by treatment with the 4x MIC of ciprofloxacin. This observation seems clinically most relevant, because a standard treatment regimen of 2x 400 mg ciprofloxacin results in concentrations in the intestinal mucosa of at least 20x MIC [12].

**Figure 2:**
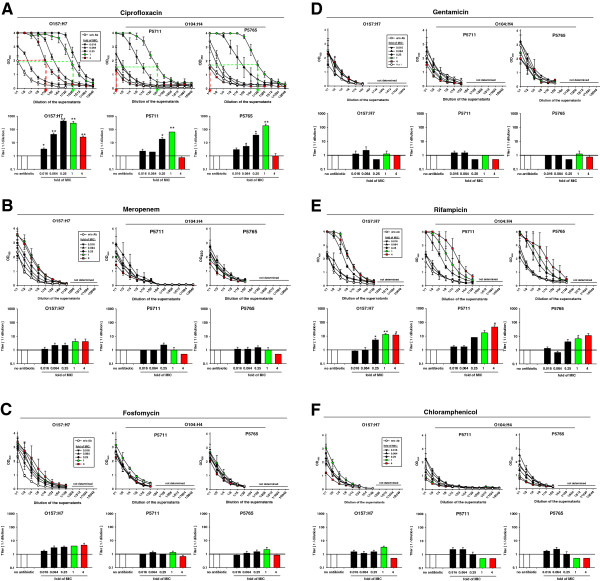
**Quantification of STX in supernatants of STEC strains O157:H7 and O104:H4 treated with various antibiotics.** The STEC cultures described in Figure 1 were harvested after 24 h of cultivation and cell-free supernatants were prepared by centrifugation and filtration. The contents of STX were determined with a commercial EIA specific for both STX1 and 2 in two-fold serial dilutions of the supernatants. For each antibiotic, in the upper part of the panel the OD of the STX-specific signal is plotted against the dilution of the supernatants. In the lower part of each panel, the STX-titers are shown which were determined in the plots of the OD as indicated exemplarily for the 1x (green dashed lines) and 4x (red dashed lines) MIC of ciprofloxacin. Briefly, from the OD-value of the undiluted sample of the untreated culture a horizontal dashed line was drawn until it intersected the plot of a given MIC. From this intersection a vertical line was drawn to determine the dilution at which the OD-value of the respective supe rnatant equaled the OD-value of the untreated control. The inverse of this dilution was defined as the STX-titer of the sample. Shown are the means and standard errors of three independent experiments. Statistical significance is indicated by asterisks: * for p < 0.05; ** for p < 0.01.

STEC O157:H7 responded to meropenem at 1x and 4x MIC with about 4-fold increased titers of STX (Figure 2B). In contrast, meropenem up to 1x MIC did not consistently increase the titers of STX2 in cultures of STEC O104:H4. Notably, 4x MIC reduced STX2 titers below those of untreated controls. Like for ciprofloxacin, a standard treatment with meropenem (1000 mg i.v.) results in 1.3 to 2.6 mg meropenem/kg colon tissue, corresponding to 30x MIC [13].

Fosfomycin at subinhibitory concentrations as well as at the 4x MIC increased the titers of STX of supernatants of strain O157:H7 up to 4-fold as compared to untreated controls, while fosfomycin did not significantly affect titers of STX2 in cultures of O104:H4 (Figure 2C). Fosfomycin has already been discussed as a risk factor increasing clinical symptoms in an outbreak of STEC O157:H7 among school children [9]. Our data document increased titers of shiga toxins in fosfomycin-treated cultures of STEC O157:H7 and, therefore, seem to support the conclusion not to treat patients infected with STEC O157:H7 with fosfomycin. However, fosfomycin does not induce the release of STX2 from STEC O104:H4 and treatment with 4x MIC even reduced STX2-titers. Thus, high doses of fosfomycin could be useful for the treatment of infections with STEC O104:H4.

Gentamicin did not enhance the release of shiga toxin from either STEC O157:H7 or O104:H4 (Figure 2D). Rifampicin at gradually increasing concentrations in the range of 0.25x to 4x MIC gradually increased the titers of STX released by both STEC O157:H7 and O104:H4 up to 64-fold of untreated controls (Figure 2E).

Chloramphenicol at 1x MIC in cultures of STEC O157:H7 increased titers about 4-fold, while a 4x MIC reduced titers below those of untreated controls (Figure 2F). In contrast, chloramphenicol at both the 1x and 4x MIC in cultures of STEC O104:H4 reduced the STX2 titers below those of untreated controls.

The determination of STX2 by EIA does only reveal the amount of immunologically detectable STX2, which is not necessarily tantamount to intact and active toxin. Thus, in order to assess the impact of antibiotics, the release of active STX2 was determined in the supernatants of fluid phase cultures of STEC O157:H7 and O104:H4 by the classical cytotoxicity assay on Vero cells. Both STX1 and 2 inhibit protein synthesis in eukaryotic cells and thereby kill Vero cells. Therefore, it has to be considered that STEC O104:H4 produces only STX2, while STEC O157:H7 produces both STX1 and 2.

**Figure 3:**
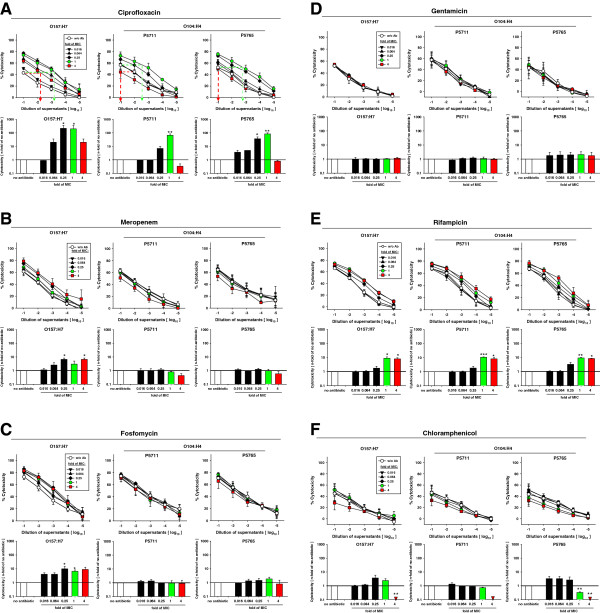
**Cytotoxic activity of supernatants of STEC strains O157:H7 and O104:H4 treated with various antibiotics.** The cell free supernatants of STEC cultures described in Figure 2 were 10-fold serially diluted and added to semi-confluent monolayers of Vero cells in microtiter plates. After incubation for 24 h, XTT-labeling reagent was added and cultures were incubated for another 24 h before measuring the viability of the Vero cells as OD_450_ of the samples. The cytotoxic activity of the supernatants was calculated as described in Methods. For each antibiotic, the cytotoxicity of the supernatants is plotted against the dilution of the supernatants in the upper part of the panel. In these plots, the effect of the antibiotics on the cytotoxicity of the supernatants was determined as the increment of cytotoxicity in comparison to untreated controls, as indicated exemplarily for the 1x and 4x MIC of ciprofloxacin by green dashed lines and red dashed lines, respectively. In the lower part of each panel the increments of the cytotoxicity are plotted for the various MIC of the respective antibiotic. Shown are the means and standard errors of three independent experiments. Statistical significance is indicated by asterisks: * for p < 0.05; ** for p < 0.01.

Concordant with the quantification of shiga toxin contents by EIA, cytotoxicity assays on Vero cells showed that treatment of STEC O157:H7 with 0.25x or 1x MIC enhanced the STX-activity of supernatants more than 100-fold (Figure 3A). Treatment of STEC O157:H7 with the 4x MIC of ciprofloxacin still increased STX activity in the supernatants more than 10-fold compared to non-treated controls. In contrast, treatment of STEC O104:H4 with 0.25x or 1x MIC of ciprofloxacin increased STX activity about 10- or almost 100-fold, respectively, compared to untreated controls. Importantly, the 4x MIC of ciprofloxacin reduced the shiga toxin activity in supernatants of STEC O104:H4 up to 10-fold compared to untreated controls.

These data confirm reports that ciprofloxacin can induce the accumulation of STX activity in the supernatants of STEC O157:H7 [3,4] and they show a similar response of STEC O104:H4 to low concentrations of ciprofloxacin. However, the dose–response of these two strains of STEC markedly varies in that 4x MIC reduces toxin activity in supernatants of O104:H4 below that of untreated controls, while the same concentration still enhances the toxin activity more than 10-fold in supernatants of strain O157:H7.

Meropenem at 0.25x and higher MIC enhanced STX activity in supernatants of strain O157:H7 up to 10-fold (Figure 3B). In contrast, meropenem at concentrations up to 1x MIC did not affect the STX activity of supernatants of strain O104:H4 and 4x MIC reduced the STX activity.

Strain O157:H7 responded to fosfomycin at concentrations of 0.25x MIC and higher with about 10-fold increased STX activity (Figure 3C). In contrast, fosfomycin did not significantly alter at any concentration the STX activity in supernatants of STEC O104:H4 cultures. Gentamicin did not affect the STX activity in the supernatants of STEC strains O157:H7 or O104:H4 at any concentration (Figure 3D). Rifampicin at 0.25x to 4x MIC increased the STX activity in the supernatants of both STEC O157:H7 and O104:H4 up to 10-fold (Figure 3E). Chloramphenicol at 1x and 4x MIC reduced the STX activity in supernatants of both strains O157:H7 and O104:H4 up to 10-fold (Figure 3F).

Taken together, the titers of STX as determined by EIA and the STX activity as measured by Vero cell cytotoxicity assay are concordant. They show that meropenem and fosfomycin at any concentration do not induce the release of STX from STEC O104:H4 and that the 4x MIC of both antibiotics even decreases the STX activity in comparison to untreated controls. Collectively, our data demonstrate that the effect of a given antibiotic upon the release of STX from a newly emerging STEC strain must not be deduced from the effect on O157:H7 or any other non-related STEC strain. Specifically, ciprofloxacin, meropenem and fosfomycin should be considered for the treatment of infections caused by strain O104:H4.

## Discussion

STEC strain O104:H4 caused the large outbreak of STEC in spring 2011 in Germany. Antibiotic treatment of STEC infected patients is generally not recommended, because enhanced release of STX from STEC O157:H7 has been reported associated with the fear of enhancing the frequency of HUS and fatalities (reviewed in [2]). This report characterizes the response of the German outbreak STEC strain O104:H4 in comparison to the prototypic STEC O157:H7. The results of this study should help to illuminate present and future medical practice.

The mechanisms of the antibiotic-induced production and release of STX by STEC have extensively been characterized *in vitro* for the most frequent STEC strain, O157:H7. Our study confirms previous reports showing enhanced STX production and release by O157:H7 in the presence of diverse antibiotics. In stark contrast, the German outbreak STEC strain O104:H4 responded to several antibiotics differently with either no release of STX or even reduced STX-titers. These data further confirm and extend previous reports that the release of STX by STEC in response to antibiotics is highly dependent on the strain of STEC and the concentration of the antibiotic [3,4].

For this study, two randomly picked different isolates, P5711 and P5765, of E. coli O104:H4 were used that were isolated from two independent patients at the Medical Center of Cologne University during the German outbreak of STEC O104:H4 in spring 2011. It should be noted that these isolates responded highly concordant to antibiotic treatment as it should be expected due to the assumed clonal origin of pathogenic microorganisms during a defined outbreak.

The impact of antibiotics on STX release merits further consideration. Despite an early induction of STX2-transcripts, meropenem does not enhance the release of STX from STEC O104:H4. The 4x MIC of meropenem even decreases STX titers and activity in supernatants of O104:H4. Since after i.v. application of meropenem peak concentrations in the relevant tissues are reached within about 1 h [13], the observed moderate induction of STX2-transcripts should not be clinically relevant. Indeed, our data suggest that meropenem is safe for the treatment of STEC O104:H4. Similarly, ciprofloxacin at concentrations equal to or beyond 4x MIC reduces the release of STX2 by STEC O104:H4 below that of untreated controls and therefore should be a safe therapeutic option against this STEC strain. These conclusions are of clinical relevance because with standard doses of either meropenem [13] or ciprofloxacin [12] concentrations far beyond the 4x MIC are achieved in humans within 1 h.

The antibiotics fosfomycin, gentamicin and chloramphenicol also appear to be suited to treat patients infected with STEC O104:H4 without increasing the release of STX. This means that there are several well-established antibiotics at hand for the treatment of infections with STEC O104:H4. Since inhibitory concentrations of these antibiotics can be achieved in patients rapidly, treatment with these substances would greatly diminish the number of, if not eradicate the bacteria and thereby prevent the sustained production and release of STX.

Previous recommendations to refrain from antibiotic treatment of STEC were not only deduced from *in vitro* data [3,4]. They were also drawn from clinical observations of more frequent and more severe symptoms of STEC infection up to increased frequencies of fatalities after treatment with antibiotics (reviewed in [2]). However, those *in vitro* as well as *in vivo* studies have to be interpreted cautiously with regard to the specific experimental conditions or to the particular STEC outbreaks. Some *in vitro* studies addressed the response of STEC only to subinhibitory concentrations of antibiotics [3,4]. A rationale for this may have been the consideration that in the beginning of antibiotic therapy, the STEC may be exposed to such low concentrations of antibiotics. However, after application of standard antibiotic doses to humans, rapid achievement of high tissue concentrations within 1 h has been reported e.g. for ciprofloxacin [12] or for meropenem [13] more than 20 or 10 years ago, respectively.

Published clinical studies are mostly retrospective studies rather than well-controlled, blinded studies which is due to the unexpected outbreaks of STEC. As a consequence, they allow only correlative conclusions rather than revealing causative mechanisms. One carefully designed prospective study [14] suffered from its small sample size as reported in a recent metaanalysis [15]. Other clinical studies have individual limitations depending on the specific conditions of the respective outbreaks. For example, Dundas et al. report about the central Scotland outbreak of STEC O157:H7 in 1996 [8]. These authors state that coincidental treatment of STEC-infected patients with antibiotics for other diseases is a risk factor for HUS and fatalities. However, such a coincidental, non-targeted antibiotic treatment cannot replace a validated, high-dose treatment specifically targeted against a defined STEC strain. Similarly, in a Japanese outbreak of STEC O157:H7 among school children, fosfomycin was used as the “most commonly prescribed antimicrobial agent in Japan” but not because it was validated as effective and safe in the treatment of this STEC strain [9].

Other clinical studies [16-18] as well as a metaanalysis [15] did not reveal a correlation between the use of antibiotics and the frequencies of the development of HUS. Consequently, in medical practice antibiotic treatment of patients infected with STEC is avoided. However, it seems unjustified to forfeit generally the antibiotic eradication of STEC and resort only to symptomatic treatment of STEC patients.

Animal studies have revealed that treatment with various antibiotics on days 1 to 3 after infection with STEC O157:H7 reduced in mice the STX levels in the blood and stool, shortened the duration of excretion of the bacteria, and all antibiotic-treated mice survived the otherwise lethal infection [19]. Similarly, mice infected with STEC O157:H7 showed enhanced survival after treatment with rifampicin alone [20] or after a sequential therapy with low dose rifampicin followed by high dose gentamicin [21].

During the final preparation of this report, Karch´s group published similar data of their concurrent study of the effects of subinhibitory concentrations of antibiotics on the German outbreak strain STEC O104:H4 with regard to the induction and release of STX [22]. In both studies, almost identical responses of STEC O104:H4 to the antibiotics meropenem, fosfomycin, gentamicin, rifampicin, and chloramphenicol were observed. At the first glance, the responses of both the outbreak strain O104:H4 and the reference strain O157:H7 seemingly differs somewhat between both reports. However, these differences are apparently due to differences in the experimental conditions applied by each group. Among these are (i) different bacterial densities at the start of antibiotic treatment (OD_600_ of 0.5 in Bielaszewska´s study versus 1x10^8^ cells/ml (corresponding to an OD_600_ of 0.1 in our hands)), (ii) analysis of induction of STX2-transcripts after 15 h versus 2 h of antibiotic treatment, (iii) or incubating Vero cells in cytotoxicity assays for 72 h versus 48 h with STX2-containing supernatants. Altogether, both reports with slightly different concepts and approaches confirm each other and therefore clearly show the potential for future controlled clinical studies using antibiotic treatment of patients infected with specific STEC strains. Newly emerging outbreak strains of STEC can be rapidly tested for the release of STX in response to relevant antibiotics. STX release can be assessed by EIA, which takes only about 2 h. Thus, the results of these assays can be available already one day after the isolation of the suspected causative STEC. Our data show that the results of the EIA and of the cytotoxicity assay on Vero cells are highly concordant. Lack of STX release in response to a specific antibiotic should provide a rationale to conduct clinical studies with the required statistically significant power that provide definitive answers to burning questions as to the potential of antibiotics to eradicate STEC, to diminish the length of carrier status, and to attenuate the development of HUS.

## Conclusions

This study suggests that there is a realistic chance for antibiotic treatment of patients in future outbreaks of STEC. Prerequisite is a rapid characterization of the respective epidemiologic EHEC strain with regard to its release of STX in response to specific antibiotics. Those antibiotics that do not enhance the release of STX should be tested in well-controlled clinical studies following the principle to treat persons as soon as possible with as high as possible doses to eradicate the STEC and thereby prevent further production and release of STX.

## Methods

### Bacteria strains

The isolates P5711 and P5765 of STEC O104:H4 were isolated from stool specimen of two HUS patients using standard diagnostic procedures at the Medical Center, University of Cologne, during the German STEC outbreak in spring 2011. According to the Helsinki Declaration, these bacteria cannot be defined as identifiable human material so that their use does not require a specific ethical approval. The reference STEC O157:H7, strain EDL933 [11] was provided by the Nationales Referenzzentrum für Salmonellen und andere Enteritiserreger, Robert Koch-Institut, Bereich Wernigerode. As an STX negative control, the *E. coli* strain ATCC 25922 was used.

### Strain typing

P7511 and P5765 were typed for the presence of STX1, STX2 by the method of Sharma et al.[23]. The presence of the following genes was determined by PCR followed by DNA probe hybridization: intimin (*eae*), *E. coli* heat labile enterotoxin (LT), invasin (*ipa*H), EAEC-heat-stable enterotoxin (EAST1), pAA virulence plasmid (*aat*A). To confirm the association of the clinical isolates with the outbreak, the recently published multiplex PCR was applied [10]. The minimal inhibitory concentrations (MIC) for ciprofloxacin, meropenem, fosfomycin, gentamicin, rifampicin, and chloramphenicol, and the ESBL phenotype were determined by E-test (AB-Biodisk).

### Induction of STX expression in liquid culture

Starter cultures (5 ml) of STEC P5711, P5765, and O157:H7 and of *E.coli* ATCC 25922, were inoculated in L-broth from single colonies on McConkey agar. After 6 hours of incubation at 37°C with vigorous shaking, 200 μl of the starter culture were inoculated into 100 ml of L-broth. After overnight incubation at 37°C with shaking, optical densities were determined and appropriate volumes were inoculated into fresh broth (5 ml L-broth) with or without antibiotics to achieve densities of 1 x 10^8^ bacterial cells/ml, i.e. the "induction cultures". Immediately after seeding, the colony forming units of these induction cultures were determined by plating serial dilutions on solid media.

The induction cultures were incubated without or with the antibiotics ciprofloxacin, meropenem, fosfomycin, gentamicin, rifampicin, or chloramphenicol at the 4x, 1x, 0.25x, 0.064x, or 0.016x minimal inhibitory concentration (MIC) determined for STEC P5711 and P5765 for 24 hours at 37°C with vigorous shaking. Subsequently, cultures were centrifuged and supernatants were filtered through 0.45 μm filters (Millipore) and stored in aliquots at -20°C.

### Quantitative RT-PCR for STX2

To determine the transcriptional induction of STX-encoding genes, 200 μl of the induction cultures were drawn two hours after start of the cultures. Total RNA was isolated (RNeasy Mini Kit, QIAGEN) and stored at −80°C. An 8 μl aliquot of each RNA extraction was transcribed into cDNA using random hexamers as primer according to the manufacturer’s instructions (SuperScript III First-Strand Synthesis System for RT-PCR, Invitrogen). cDNA was stored at −20°C until further use. Quantitative PCR was set up using the hydrolysis probe assay for STX2 as described by Sharma et al. for detection of STX2 genomic DNA [23]. Each cDNA was run in duplicate together with a dilution series of an STX2 plasmid standard on an LightCycler 480 realtime PCR machine with quantification software. Copy numbers of STX2 transcripts were calculated against the STX2 plasmid standard.

### Quantification of STX in STEC supernatants by EIA

The contents of STX in the filtered supernatants of the bacterial cultures incubated with or without antibiotics were determined by a solid phase enzyme immunoassay (EIA) that detects both STX 1 and 2 (ProSpecT, REMEL, Lenexa, KS, USA). To assess the quantitative effect of antibiotics on the release of STX, 2-fold serial dilutions of the supernatants were subjected to the EIA. The STX titer of a given supernatant was defined as the reciprocal dilution at which the optical density (OD) of the sample equaled the OD of the undiluted supernatant of the respective bacteria cultured without antibiotics.

### STX activity in STEC supernatants

The toxin activity of STX in supernatants of bacterial cultures was determined by a Vero cell cytotoxicity assay modified from an assay of Gentry et al. [24]. Briefly, 100 μl of Vero cell suspensions were seeded in a 96-well plate at a density of 1.6 x 10^5^ cells/ml and grown for 24 h at 37°C in 5% CO_2_ atmosphere. Subsequently, 100 μl of 10-fold serial dilutions of the filtered supernatants of bacterial cultures were added to the cultures. After incubation for another 24 h, XTT labelling reagent was added to the wells according to the instructions of the manufacturer (Roche Applied Science, Mannheim, Germany) for 24 h before the OD_450_ of individual wells was measured with a Tecan infinite M1000 instrument. Vero cell cultures without bacterial supernatants and cell-free samples of media alone with XTT-reagent were included to determine the values of the maximal cell viability and the background, respectively. From these readings, the values of cytotoxicity were calculated by the formula:

(1) $$\%\text{Cytotoxicity}=\left(1-\left[\left({\text{OD}}_{\text{sample}}-{\text{OD}}_{\text{background}}\right)/\left({\text{OD}}_{\text{max}}{\;}_{\text{viability}}-{\text{OD}}_{\text{background}}\right)\right]\right)\times 100$$

### Statistical analysis

Statistical significance was assessed by applying Student´s paired *t*-test. The levels of significance are indicated by asterisks in the figures.

## Competing interests

The authors declare that they have no competing interests.

## Author´s contributions

DC did the bacterial cultures, harvested the supernatants, performed the EIA, established and performed the cytotoxicity assays. RW did the bacterial cultures, harvested the supernatants, and quantified the transcriptional response of bacteria. MW established conditions for the bacterial cultures, harvested the supernatants. GP genotyped the bacteria and quantified the transcriptional response of bacteria. OU coordinated the study, established the cytotoxicity assay, analysed data and wrote the manuscript. MK designed the study, analysed data and wrote the manuscript. All authors read and approved the final manuscript.
